# Timing of noninvasive ventilation failure: causes, risk factors, and potential remedies

**DOI:** 10.1186/1471-2466-14-19

**Published:** 2014-02-13

**Authors:** Ezgi Ozyilmaz, Aylin Ozsancak Ugurlu, Stefano Nava

**Affiliations:** 1 Cukurova University Faculty of Medicine Department of Pulmonary Disease, Adana, Turkey; 2 Baskent University Faculty of Medicine Department of Pulmonary Disease, İstanbul, Turkey; 3 Department of Specialistic, Diagnostic and Experimental Medicine (DIMES), Respiratory and Critical Care, University of Bologna, Sant'Orsola Malpighi Hospital building #15, Alma Mater Studiorum, via Massarenti n.15, Bologna 40185, Italy

**Keywords:** Noninvasive ventilation, Treatment failure, Respiratory insufficiency

## Abstract

**Background:**

Identifying the predictors of noninvasive ventilation (NIV) failure has attracted significant interest because of the strong link between failure and poor outcomes. However, very little attention has been paid to the timing of the failure. This narrative review focuses on the causes of NIV failure and risk factors and potential remedies for NIV failure, based on the timing factor.

**Results:**

The possible causes of immediate failure (within minutes to <1 h) are a weak cough reflex, excessive secretions, hypercapnic encephalopathy, intolerance, agitation, and patient-ventilator asynchrony. The major potential interventions include chest physiotherapeutic techniques, early fiberoptic bronchoscopy, changing ventilator settings, and judicious sedation. The risk factors for early failure (within 1 to 48 h) may differ for hypercapnic and hypoxemic respiratory failure. However, most cases of early failure are due to poor arterial blood gas (ABGs) and an inability to promptly correct them, increased severity of illness, and the persistence of a high respiratory rate. Despite a satisfactory initial response, late failure (48 h after NIV) can occur and may be related to sleep disturbance.

**Conclusions:**

Every clinician dealing with NIV should be aware of these risk factors and the predicted parameters of NIV failure that may change during the application of NIV. Close monitoring is required to detect early and late signs of deterioration, thereby preventing unavoidable delays in intubation.

## Review

The utilization of noninvasive mechanical ventilation (NIV) has become one of the most important developments in the field of mechanical ventilation over the past two decades. The use of NIV during acute respiratory failure (ARF) has increased since the late 1990s for all diagnoses, including patients with and without chronic obstructive pulmonary disease (COPD), regardless of the supporting evidence for the later [1].

NIV failure has been defined as the need for endotracheal intubation (ETI) or death [2]. Its rate greatly varies between 5 and 60%, depending on numerous factors, including the cause of ARF [3,4]. Unsuccessful NIV was found to be independently associated with death, especially in patients with de novo ARF [5]. This may indicate the need for caution with regard to the application of NIV and for close monitoring to switch promptly to ETI when necessary.

**Figure 1:**
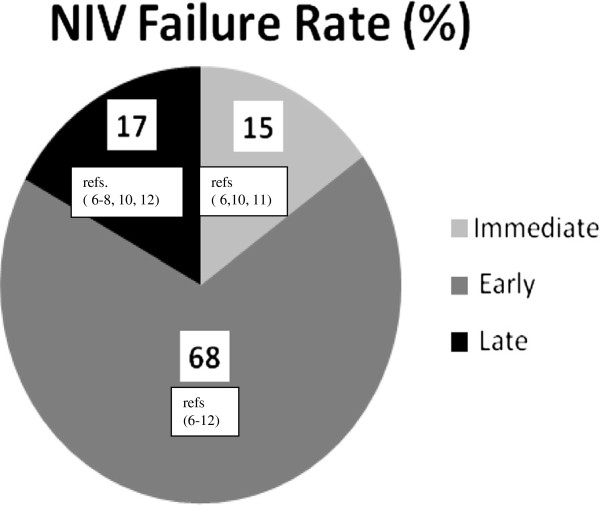
Mean NIV failure rates based on timing according to the data of randomised controlled trials (6–12).

Several investigators have tried to assess the best predictors of NIV failure [6-12]. However, to the best of our knowledge, despite the rather extensive literature in the NIV field, there is only one paper, published 10 years ago, summarizing the evidence for the risk factors for NIV failure, and no studies of the timing of the failure [13]. Based on data from randomized controlled trials (RCTs), three temporal moments were identified: 1) immediate failure (within minutes to <1 h), 2) early failure (1 to 48 h), and 3) late failure (after 48 h) (Figure 1) [6-12]. The purpose of this narrative review is to illustrate the main patient-related predictors or risks factors of immediate, early, and late failure. We also discuss possible remedies to avoid ETI and nonpatient-related risk factors.

### Patient related risk factors

#### Immediate NIV failure

Immediate NIV failure refers to failure within minutes and not beyond the first hour. Excluding patients with contraindications for NIV use (Table 1) [4], about 15% of all NIV failures were defined as “immediate” in RCTs, irrespective of the underlying causes of ARF [6,10,11]. Predictors of failure in this period have never been systematically analyzed. The causes of NIV failure and possible remedies are reviewed below (Table 2).

**Table 1:** **Indications and contraindications for NIV in acute care**[4,16,17,35]


**Indications:**	
**A- Gas exchange:**	
•	Acute or acute on chronic ventilator failure (best indication), PaCO_2_ > 45 mmHg
•	Ph < 7.35
•	Hypoxemia (use with caution), PaO_2_/FIO_2_ ratio < 200
**B- Bedside observations:**	
•	Increased dyspnea- moderate to severe
•	Tachypnoea (24 breaths per minute in obstructive, >30 per minute in restrictive)
•	Signs of increased work of breathing, accessory muscle use, and abdominal paradox
**Absolute contraindications:**	
•	Cardiac or respiratory arrest
•	Unable to fit mask
**Relative contraindications:**	
•	Non-respiratory organ failure (severe encephalopathy with GCS < 10, severe upper gastrointestinal bleeding, hemodynamic instability or unstable cardiac arrthythmia)
•	Inability to cooperate/protect the airway
•	Inability to clear respiratory secretions
•	High risk of aspiration
•	Recent facial surgery, trauma, or deformity
•	Upper airway obstruction

NIV: Noninvasive ventilation, GCS: Glasgow coma scale.

**Table 2:** The risk factors and suggestions for the management of NIV failure based on timing

**Time**	**Risk factors**	**Suggested interventions**	**References**
**Immediate**	1. Weak cough reflex and/or excessive secretions	1a. Manual or mechanic chest physiotherapeutic techniques; b. Early fiberoptic bronchoscopy.	[14-24]
	2. Hypercapnic encephalopathy and coma	2a. Set a back-up rate ~ 15 b/min and/or use PCV; b. Decrease the F_İ_O2 level.	[16,25-27]
	3. Intolerance and psychomotor agitation	3. Judicious sedation	[14,28-32]
	4. “Fighting with the machine”: Patient-ventilator asynchrony	4a. Closely monitor ventilator waveforms; b. Judicious sedation; c. Use a ventilator with an NIV platform; d. Change ventilatory parameters; e. Minimize air-leaks.	[33,34]
**Early Hypoxemic ARF**	1. Baseline ABG and inability to correct gas exchange (P/F < 150)*		[4] ([36,38,39,42]*])
	2. Baseline severity scores (SAPS II >35)*		[1,5,36]*,[37]*,[39,43,49]*-[51]
	3. The presence of ARDS/pneumonia/sepsis/multiorgan failure (OR: 4-28)*		[36]*,[40,41,49,50,52]*-[54]
	4. Increased respiratory rate (>25 breaths/min)*		[51]*,[53,55]*,[56]
	5. Miscellaneous: Delay between admission and NIV use, Number of fiberoptic bronchoscopes performed, Duration of NIV use, Increase in radiographic infiltrates within the first 24 hours, Causal diagnosis (as ‘de novo’)		[1,36,43,51,53,57]
**Hypercapnic ARF**	1. Baseline ABG and inability to correct gas exchange (pH < 7.25)*		[3]*,[6,12,14,58-60,62,63]
	2. Increased severity of disease		[3,12,14,15,59,62,64]
	3. Increased respiratory rate (>35 breaths/min, OR for baseline and after 2 hours of NIV: 2.66 and 4.95)*		[3]*,[6,15,58,59]
	4. Mixed indices:		
	GCS, APACHE II score, respiratory rate and pH		[3]
	Respiratory rate, random glucose level and APACHE II		[66]
	Anemia and World Health Organization Performance Status (WHO-PS)		[67]
	5. Miscellaneous: Poor nutritional status, Increased heart rate, Higher baseline C-reactive protein/white blood cell count, Lower serum potassium, Airway colonization by non-fermenting gram-negative bacilli		[63,68,69]
**Late**	1. Sleep disturbance	1a. Improve quality of sleep; b. Avoid excessive noise and light in the unit	[70]
	2. Functional limitation		[2]
	3. Possible initial improvement in pH	3a. Close and continuous monitoring of vital parameters; b. Repeat frequent ABGs during NIV, even when gas exchange reach a satisfactory value	[2,64]
	4. Hyperglycemia		
		4. Check glucose levels	[2]

Odd Ratio (OR) or absolute “predictive” values for some parameters are reported from the studies quoted in the references and marked with * from other references.

##### Weak cough reflex and/or excessive secretions

NIV does not allow direct access to the airways. A weak cough reflex leading to inefficient clearance of excessive secretions from airways is a common cause of immediate NIV failure [14,15]. The inability to spontaneously remove secretions is considered a relative contraindication for NIV, especially in patients with impaired consciousness and depressed cough [16,17]. Some data indicate that specific “manual” or “mechanical” physiotherapeutic techniques may improve mucociliary clearance during NIV and that NIV can still be used in those circumstances [18,19]. Intrapulmonary percussive ventilation (IPV) is a technique that delivers small bursts of high-flow respiratory gas at high rates for mobilization of secretions [20]. Two clinical studies demonstrated that IPV used before or in combination with NIV may reduce the risk of ETI in COPD patients with difficulties removing secretions [19,21]. Early fiberoptic bronchoscopy is another potential intervention that can be used to minimize the burden of respiratory secretions. In a matched case–control study, early suction of secretions performed during NIV was shown to be feasible and safe [22]. However, in a recent prospective multicenter study, ventilatory support needed to be increased after fiberoptic bronchocopy in 35% of patients with hypoxemic respiratory failure [23].

In conclusion, for patients with a weak cough reflex or excessive secretions, cautious secretion management during NIV use might be advisable before NIV is declared as failed [4,24].

##### Hypercapnic encephalopathy and coma

Hypercapnic encephalopathy (HES) is often considered a cause of immediate NIV failure because of poor compliance due to confusion and/or agitation. Additionally, it is viewed as a relative contraindication because of the increased risk of aspiration [16].

A number of studies clearly demonstrated that cautious application of NIV can be attempted in patients with HES by an experienced team to achieve a fast reduction of PaCO_2_ and to re-institute the conditions for a successful NIV attempt [25,26]. The risk of aspiration has been shown to be minimized by the rapid improvement of neurological status under NIV and NIV failure rates were reported to be comparable among patients with and without HES [25]. The use of a relatively high back-up rate and/or pressure control ventilation may also help to “capture” the patient better [26]. Another key factor in patients with HES is the rebound effect of high-fractionated oxygen (F_İ_O_2_) on the PaCO_2_ and pH, known as the “Haldane effect” [27]. This effect can be prevented by a simple intervention: decreasing the F_İ_O_2_ level.

##### Intolerance and psychomotor agitation

Patient tolerance has been shown to be critical for NIV success, especially in the first few minutes while the patient adapts to this “new mode” of breathing [14]. Interfaces can lead to intolerance. However, the role of these interfaces will be discussed later because they are not directly related to the patient.

The use of judicious sedation may be valuable to achieve a sedation level that keeps the patient awake, easily arousable, and comfortable. Ideal sedatives should be short acting and have no significant effects on respiratory drive and hemodynamics [28-30]. Remifentanil, an opioid with an elimination half-life less than 10 min, provides rapid onset of sedation and easy arousal. Two pilot studies performed in patients who initially failed an NIV trial due to intolerance and therefore met ETI criteria showed that the administration of remifentanil removed the need for intubation in most of the patients [31,32]. A pilot study showed that a safe and satisfactory level of sedation during NIV could be achieved with dexmedetomidine, an alpha-2-adrenoceptor agonist [30]. Compared to midazolam, dexmedetomidine led to a more desired level of awaking sedation, and it shortened the duration of mechanical ventilation and ICU stays in patients with acute cardiogenic pulmonary edema.

Although sedation has been suggested as a remedy for intolerance, the “real life experience” shows that, only a minority of physicians use sedation and analgesia during NIV as a routine and it is usually without a specific protocol [29].

It has to be kept in mind that oversedation during NIV can be potentially dangerous. Thus, close monitoring with evaluation of arterial blood gas (ABG), cardiopulmonary and ventilator parameters, adverse events, and the level of sedation is mandatory.

##### Fighting with the machine: patient-ventilator asynchrony

Asynchrony has rarely been cited as a direct cause of NIV immediate failure. However, indirect evidence suggests that this may be the case [33]. Asynchrony can easily be detected by a physical examination (e.g., number of spontaneous breaths vs. ventilator-delivered breaths, accessory muscle use) of the patient and symptoms (e.g., dyspnea). Two main causes of asynchrony are a high level of ventilator support and an increased number of leaks.

A number of strategies can be implemented to avoid “gross asynchronies,” such as optimization of ventilator settings using the screen ventilator waveforms, adjusting trigger sensitivity, increasing positive end-expiratory pressure, minimizing leaks, using different modes or more sophisticated ventilators [33]. New modes of ventilation, such as neutrally adjusted ventilator assist, have been documented to reduce asynchrony [34].

#### Early NIV failure

Nearly 65% of NIV failures occur within 1–48 h of NIV use (Figure 1), [6-8,10,12]. This time interval has received more attention in assessments of predictors of failure. As most studies have focused on NIV use in either hypercapnic or pure hypoxic ARF, these two conditions will be considered separately. Rather than giving possible solutions, as in the previous section (Section 1), we will discuss the most reasonable clinical decision to be applied.

##### Hypoxemic respiratory failure

Hypoxemic ARF can be the end point of several pathologies, including pneumonia, acute respiratory distress syndrome (ARDS), and cardiogenic pulmonary edema (CPE), each of which acts through different mechanisms (e.g., shunting, ventilation/perfusion mismatch, or diffusion limitation). Robust RCTs of the utilization of NIV for hypoxemic ARF are scarce, explaining the absence of specific recommendations in evidence-based guidelines [35]. Therefore, it is difficult to make general statements about risk factors and predictive factors of NIV failure covering all causes of hypoxemic ARF (Table 2).

##### Baseline ABG and inability to correct gas exchange

Oxygenation impairment, as shown by a decreased ratio of PaO_2_ to FiO_2_ (P/F ratio), is one of the most well-documented risk factors and predictors of NIV failure. The outcomes of the patients are probably more dependent on the underlying cause, rather than on the baseline severity of the hypoxemia itself. A prospective multicenter study investigating variables predictive of NIV failure in 354 patients with hypoxemic ARF reported a higher ETI rate in patients with ARDS (51%) and community-acquired pneumonia (CAP) (50%) than in patients with pulmonary contusion (18%) and CPE (10%) [36]. In this study, most ETIs occurred due to the inability of NIV to correct gas exchange (62%). Although the ABG values at study entry had no predictive value, severe hypoxemia (P/F ≤146) after 1 h of NIV treatment was reported to be an independent predictor of NIV failure according to multivariate analysis [36].

### ARDS and CAP

In a group of patients with ARDS, the inability to improve the P/F ratio after 1 h of NIV use (P/F ≤175) was shown to be an independent predictor of NIV failure [37]. However, in another prospective observational study, the baseline P/F ratio (<120) was shown to be the only factor associated with NIV failure [38]. Likewise, a low P/F ratio at admission was found to be a risk factor for immune-suppressed patients with pneumonia and extrapulmonary sepsis and for patients with H_1_N_1_ pneumonia [39,40] and acute lung injury (ALI) (in this latter group with an odd ratio = 1.03, per unit decrease in PaO_2_/FiO_2_) for NIV failure [41]. P/F ratios at both baseline (around 115) and after 1 h of NIV use (around 140) were indicated as independent predictors of NIV failure in patients with severe CAP [42].

Metabolic values on ABGs, other than oxygen levels, should also be carefully assessed. The serum bicarbonate level, as well as the P/F ratio, 1 h after NIV onset was an independent predictor of NIV failure in patients with CAP and severe ARDS [43]. Metabolic acidosis was reported to be a significant predictor of unsuccessful NIV in patients with ALI [41].

### Cardiogenic pulmonary edema (CPE)

Generally, the rate of NIV failure is very low in patients with CPE. In a study of 2430 patients, the NIV success rate was 96%, and oxygen saturation was lower in the failure group [44]. Masip et al. demonstrated that acute myocardial infarction, a low pH (<7.25), a low ejection fraction (<30%), hypercapnia, and low systolic blood pressure (<140 mmHg) were independent predictors for ETI [45]. A pH level of 7.03 was shown to be a cut-off level to predict NIV success, with the highest sensitivity and specificity observed in patients with CPE [46].

To summarize, the severity of hypoxemia and acidosis and their initial responses to NIV are strong predictors of NIV outcomes. NIV should be performed very cautiously, especially in patients with a P/F < 150 and diagnosed as ARDS or CAP. Vital signs and ABGs should be monitored very closely, starting even before the “classical” 60 min frame. The initial amelioration of ABGs does not imply that NIV will be successful, and strict monitoring should be continued in the following days [36-41].

#### Baseline severity scores

Although some previous research failed to indicate any relationship between baseline severity scores (reflecting the severity of the disease) and NIV outcomes [47,48], most recent trials clearly confirmed this relationship [1,5,36,37,49].

Higher SOFA, APACHE II, and/or SAPS II scores were related with NIV failure in studies performed in either all patients with ARF or patients with hypoxemic ARF postoperatively or hypoxemic ARF due to sepsis, pneumonia or hematological malignancies [1,5,39,43,49-51]. An SAPS II score ≥34 was identified as an independent risk factor for NIV failure in hypoxemic ARF [36,37].

To avoid harmful delays in ETI, these indexes should always be evaluated in patients using NIV with these disorders. It should be kept in mind that an SAPS II score in the middle 30s is associated with a very high risk of failure.

#### Presence of ARDS/pneumonia/sepsis/multiorgan failure

As stated earlier (2.1.1), underlying disease is the major risk factor for NIV failure. De novo ARF was shown to be associated with NIV failure and subsequent ETI in all ARF patients administered NIV [1,5]. In an observational study, the presence of ARDS or CAP was identified as a risk factor for NIV failure (OR = 3.75) [36]. In an RCT, ARDS was significantly associated with ETI (OR = 28.5) [52]. These findings were also confirmed in patients with pulmonary infiltrates due to hematological malignancy or CAP in postoperative ARF [49,50,53].

Multiorgan failure and hemodynamic instability have been documented as risk factors of NIV failure in immunosuppressed patients with hypoxemic ARF [40,53]. In an observational study, 35% of patients with ALI requiring NIV were diagnosed with septic shock, and NIV failed in all the patients [41].

At present, we cannot recommend the use of NIV in patients with moderate to severe hypoxia (P/F = <200) diagnosed with ARDS or CAP or in patients with septic shock, in agreement with the statement of the surviving sepsis campaign [54].

#### Increased respiratory rate

An increased respiratory rate 1 h after NIV is a frequently reported risk factor for NIV failure in patients with ARF postoperatively or ARF due to hematological malignancies or ALI [51,53,55]. These studies suggested that an average respiratory rate >25 breaths/min on NIV is a predictor of failure because it is a surrogate of increased work of breathing. The so-called rapid shallow breathing index >105 (i.e., the ratio between the breathing frequency/tidal volume) under NIV has also been demonstrated to be independently associated with ETI (multivariate OR: 3.70) [56].

#### Miscellaneous risk factors of NIV failure

Other risk factors for NIV failure in hypoxemic ARF are the delay between admission and NIV use, number of fiberoptic bronchoscopies performed, duration of NIV use, increase in radiographic infiltrates within the first 24 h, and causal diagnosis (as de novo) of ARF [1,43,51,53]. Age has been demonstrated to be a risk factor (with a low OR) only in a minority of studies [1,36,57].

### Hypercapnic respiratory failure

The utilization of NIV in hypercapnic ARF, as well as the risk factors for its failure, has probably been more intensively studied compared to its use in hypoxic ARF (Table 2). Although hypercapnic ARF covers ARF due to neurological disorders (such as neuromuscular disorders) or other acute or chronic lung disorders (such as restrictive lung disease), most of the studies done in this field have involved patients with COPD exacerbations [3,58].

#### Baseline ABG and inability to correct gas exchange

The pH level, which is an indicator of the severity of hypercapnia, has been reported to be a critical factor in determining the success of NIV. Although some reports failed to show any relationship between baseline ABGs and the success of NIV [15,48], a large body of evidence clearly indicated that a lower baseline pH is a risk factor for NIV failure in COPD patients [3,6,12,14,58-60]. In nearly 50–60% of patients with a baseline pH of <7.25, NIV was unsuccessful [6,12]. A subgroup analysis of COPD patients with mild to moderate acidosis revealed that NIV improved patient outcomes only if the baseline pH was ≥7.30 [58].

In addition to baseline levels, pH values 1 h after the application of NIV were shown to be strong predictors of the success of NIV, with high sensitivity and good specificity (93% and 82%, respectively) [59]. In a study of more than 1000 COPD patients, Confalonieri et al. pointed out that a pH <7.25 after 1 h of NIV use was associated with an increased risk of failure and that the risk of failure was even greater than when the pH levels were <7.25 at admission [3].

For the above mentioned reasons, we do not recommend routine use of NIV in patients with a pH <7.25 outside a “protected” environment [16,17]. However, recently, NIV has been offered as an effective treatment option for patients with severe acidosis due to COPD, even if performed in a respiratory ward [61]. In this study, NIV improved pH and PaCO_2_ to the same extent in two groups of patients with mild acidosis and severe acidosis (pH <7.25) and overall survival rate was also comparable. However, the study’s findings should be interpreted cautiously. RCTs are required to confirm that NIV can be safely applied in patients with severe acidosis outside step-down units or ICUs.

Risk factors and predictors of NIV failure were also assessed in non-COPD patients with hypercapnic ARF. A low P/F ratio (on average <200), higher PaCO_2,_ and a lower pH after 1 h of NIV were reported as independent predictors of NIV failure in a subset of heterogeneous patients, including those with bronchiectasis and pulmonary tuberculosis sequelae [62,63].

#### Increased severity of disease

The relationship between NIV failure and the severity scores, including APACHE II and SAPS II, has been documented in a number of reports. Several researchers found an association [3,12,14,15,59,62,64], whereas others failed to find any association [5,48]. Interestingly, in a study of more than 500 patients, although a high SAPS II was a strong indicator of NIV failure and death in hypoxic ARF, this was not the case for hypercapnic ARF (OR = 3.05 vs. 1.17, respectively) [5]. Therefore, in this latter population, the presence of acute or chronic comorbidities may be stronger risk factors for NIV failure than the severity indices [65].

#### Increased respiratory rate

An initial high respiratory rate and its reduction after 1 h of NIV have been shown to be associated with successful NIV outcomes in COPD patients [6,15,58,59]. A respiratory rate of 30–34 and ≥35 breaths/min at admission were demonstrated to lead to NIV failure, with an OR of 1.83 and 2.66, respectively, whereas the ratios increased to 2.67 and 4.95, respectively, for the same breathing frequency after 2 h of NIV [3].

#### Mixed indices

Some investigators have suggested using mixed indices to improve the probability of the prediction of NIV failure. A risk stratification chart of NIV failure demonstrated that COPD patients with a Glasgow Coma Scale (GCS) <11, an APACHE II score ≥ 29, a respiratory rate ≥30 breaths/min, and a pH <7.25 at admission had a risk of failure >70% [3]. The risk increased up to 95% for the same parameters after 2 h of NIV therapy [3]. The prediction of NIV success was 97% with a combination of a respiratory rate <30 bpm and glucose <7 mmol/L [66]. Anemia and a World Health Organization performance status (WHO-Performance Status) score ≥3 were also shown to be significant predictors of mortality and NIV failure [67].

Thus far, the risk chart developed by Confalonieri et al. [3] is probably the best mixed index for predicting NIV outcomes with reasonable accuracy.

#### Miscellaneous

Poor nutritional status (i.e., a low BMI), a high white blood cell count, low serum potassium, and an increased heart rate are additional risk factors for NIV failure [63,68]. Two additional issues may merit specific consideration. Old age has never been shown to be a “negative” variable in determining NIV outcomes, and older patients with hypercapnic ARF may respond even better than younger ones to NIV [69]. The combination of ARF and pneumonia is probably one of the strongest determinants of NIV failure [59], but this has never been extensively studied, as most of the RCTs excluded a priori these patients.

### Late NIV failure

Although the definition of late NIV failure has not been standardized; it is usually defined as failure that occurs 48 h after initiation of NIV, following an initial successful response. Late NIV failure has received less attention and has been studied mainly in hypercapnic ARF. [2,6-8,10,12,64,70]. Actually, it occurs in a considerable subset of patients (about 15% of NIV failures) (Figure 1).

The occurrence of late failure in COPD patients admitted with hypercapnic ARF to ICUs when NIV was used >24 h was found to be associated with functional limitation before admission, the presence of hyperglycemia, and a lower pH at admission [2]. PaCO_2_ and pH values improved gradually and similarly within the first 24 h in both success and late failure groups [2]. This is of particular importance because initial good responses to NIV may decrease attention and monitoring by clinicians in the following hours [2,64]. During a hospital stay, pneumonia was more frequently observed as a complication in a late failure group compared to a success group (12.9% vs. 0%) [2]. It is logical that the occurrence of infectious complications and/or multiple organ failure may result in late NIV failure, but such risk factors have never been addressed in a trial. The mortality of a late failure group was extremely high compared to a successful group in another study (68 vs. 0%) [2]. In a recent study, sleep disturbance (classified as an abnormal electroencephalographic pattern, greater circadian sleep-cycle disruption, and less nocturnal rapid eye movement sleep) and increased delirium during an ICU stay were also associated with late NIV failure in hypercapnic patients [70].

NIV patients should be continuously monitored (including their sleep patterns and state of delirium), even if their initial clinical and ABG responses are good because late NIV failure can lead to high mortality.

### Non-patient related risk factors

The timing of the application of NIV is a critical factor. A longer delay between admission and NIV use was shown to be an independent risk factor for NIV failure in patients with hematological malignancy and hypoxemic ARF, probably due to the progression of the underlying disease [53]. Therefore, early use of NIV is recommended. It is also critical not to unduly delay the decision to intubate a patient with failed NIV, because the risk of unanticipated respiratory or cardiac arrest could lead to increased morbidity and mortality.

The location of the NIV therapy is another important determinant in the success of NIV. There are advantages and disadvantages of different locations (including ICUs, step-down units, wards, and emergency care) for NIV application, and these have been discussed in detail elsewhere [71]. The decision about where to perform NIV should be based on matching the capabilities of the units and teams with the patient’s clinical severity and the need for monitoring.

The experience and the skills of the staff are other key components of NIV success. One study suggested that training in NIV implementation is an important factor in reducing nasocomial infections and improving survival in critically ill patients with COPD and ACPE [72]. Another found that improvements in skill with time may explain the decreased time spent by nurses at the bedside of patients today compared to data reported 20 years ago [73].

The choice of ventilator is crucial in NIV success in the acute setting, with inadequate equipment leading to poor tolerance and excessive air leaks being documented as a barrier to NIV use [74]. On average, dedicated NIV platforms perform better than ICU ventilators using the NIV algorithm [75]. In particular, the synchrony between the machine and the patient is better with dedicated NIV platforms [75].

Although much attention has been paid to the development of new interfaces to increase tolerance and patient comfort, mask intolerance remains a major cause of NIV failure [32]. An oronasal mask is generally the most commonly preferred one in ARF, followed by nasal masks, helmets, and mouthpieces. There are various advantages and disadvantages of these interfaces. In the case of poor tolerance, a wise choice may be the application of the so-called “rotating” strategy proposed by Hilbert et al. [76].

Some authors concluded that humidification during NIV for ARF is controversial and that the effect of humidification on the success of NIV is unclear [77]. However, heated humidification is recommended to minimize the work of breathing and to maximize PaCO_2_ clearance, with less dead space than ventilators with heat and moisture exchangers.

## Conclusions

Risk factors and predictors of NIV failure are numerous. They differ between hypercapnic and hypoxemic patients and according to the timing of the failure. Every physician dealing with NIV should be aware of these risk factors and closely monitor each patient for their presence or development to achieve a good response and to improve the prognosis. If a patient fails to improve sufficiently, prompt ETI should be performed without a delay because there is an increased risk of morbidity and mortality with ETI after failed NIV. A satisfactory initial NIV attempt is not always a marker of a good outcome. Late NIV failure may occur in up to 15% of patients in whom the initial NIV attempt was satisfactory.

## Competing interests

The authors declare that they have no competing interests.

## Authors’ contributions

EO, AOU and SN designed the study. EO and AOU collected the data involved in drafting the manuscript. SN revised the manuscript critically for significant intellectual content. All of the authors have given final approval of the version to be published.

## Pre-publication history

The pre-publication history for this paper can be accessed here:

http://www.biomedcentral.com/1471-2466/14/19/prepub
